# Menadione Suppresses Benzo(α)pyrene-Induced Activation of Cytochromes P450 1A: Insights into a Possible Molecular Mechanism

**DOI:** 10.1371/journal.pone.0155135

**Published:** 2016-05-11

**Authors:** Yulia A. Sidorova, Maria L. Perepechaeva, Elena N. Pivovarova, Arkady L. Markel, Vyacheslav V. Lyakhovich, Alevtina Y. Grishanova

**Affiliations:** 1 Institute of Molecular Biology and Biophysics, Novosibirsk, Russia; 2 Federal research center Institute of Cytology and Genetics, Siberian Branch of Russian Academy of Sciences, Novosibirsk, Russia; College of Tropical Agriculture and Human Resources, University of Hawaii, UNITED STATES

## Abstract

Oxidative reactions that are catalyzed by cytochromes P450 1A (CYP1A) lead to formation of carcinogenic derivatives of arylamines and polycyclic aromatic hydrocarbons (PAHs), such as the widespread environmental pollutant benzo(α)pyrene (BP). These compounds upregulate CYP1A at the transcriptional level via an arylhydrocarbon receptor (AhR)-dependent signaling pathway. Because of the involvement of AhR-dependent genes in chemically induced carcinogenesis, suppression of this signaling pathway could prevent tumor formation and/or progression. Here we show that menadione (a water-soluble analog of vitamin K_3_) inhibits BP-induced expression and enzymatic activity of both CYP1A1 and CYP1A2 *in vivo* (in the rat liver) and BP-induced activity of CYP1A1 *in vitro*. Coadministration of BP and menadione reduced DNA-binding activity of AhR and increased DNA-binding activity of transcription factors Oct-1 and CCAAT/enhancer binding protein (C/EBP), which are known to be involved in negative regulation of AhR-dependent genes, *in vivo*. Expression of another factor involved in downregulation of CYP1A—pAhR repressor (AhRR)—was lower in the liver of the rats treated with BP and menadione, indicating that the inhibitory effect of menadione on CYP1A is not mediated by this protein. Furthermore, menadione was well tolerated by the animals: no signs of acute toxicity were detected by visual examination or by assessment of weight gain dynamics or liver function. Taken together, our results suggest that menadione can be used in further studies on animal models of chemically induced carcinogenesis because menadione may suppress tumor formation and possibly progression.

## Introduction

Cytochromes P450 of the 1A subfamily (CYP1A1 and CYP1A2) metabolize most common environmental pollutants: polycyclic aromatic hydrocarbons (PAHs) (such as benzo(α)pyrene (BP), BP-like compounds) and arylamines. These enzymes catalyze all reactions of PAH biotransformation including the ones generating highly reactive molecules that exert carcinogenic and mutagenic effects on the cells [1, 2].

PAHs induce CYP1A expression via a ligand-activated transcription factor aryl hydrocarbon receptor (AhR) belonging to the family of bHLH/PAS (basic helix-loop-helix/Per-ARNT-Sim) transcription factors. Inactive AhR is located in the cytoplasm, in complex with other proteins. Ligand-bound AhR moves to the nucleus and dimerizes with Ah receptor nuclear translocator (ARNT). The resulting heterodimer binds to xenobiotic responsive elements (XREs) in the enhancers of AhR-dependent genes and enhances their transcription [2].

Negative regulation of CYP1A has been studied less thoroughly. CYP1A suppression can be mediated by AhR repressor (AhRR), which competes with AhR for binding to ARNT and forms the heterodimer AhRR–ARNT; the latter binds to XRE but fails to upregulate gene expression. In addition, transcription factors Oct-1 and C/EBPβ, whose binding sites are located in the upstream region of the *CYP1A1* gene, can repress expression of *CYP1A1* independently of AhR [3, 4].

For a long time, the only known inducers of CYP1A have been PAH-like compounds [5]. In the last decades, however, numerous structurally diverse compounds were discovered that weakly or moderately activate CYP1A: flavonoids, indoles [6, 7], and synthetic retinoids [8], benzimidazole drugs [9], quercetin, resveratrol, and curcumin [10]. Some of them were shown to bind to AhR [6–8]. Other compounds may activate AhR-dependent signaling and *CYP1A* expression indirectly [9, 10].

Many of these weak inducers can suppress PAH-induced activation of CYP1A [11–16] by different mechanisms, for instance, competition for the active center of AhR [12], inhibition of phosphorylation of AhR and/or ARNT [17], or activation of transcriptional suppressors of CYP1A1 [4]. In many cases, however, molecular mechanisms of downregulation of PAH-induced CYP1A1 activity by weak inducers have yet to be elucidated.

Because most if not all negative effects of PAHs are mediated by AhR and AhR-dependent genes [18], the suppression of AhR activity and downregulation of its target genes may be used to reduce the adverse effects of PAHs and to control chemically induced carcinogenesis. Indeed, experimental animals lacking AhR or CYP1A1 were shown to be resistant to the toxic and carcinogenic effects of classical AhR ligands [19–22]. In addition, many of the substances interfering with the action of “classic” CYP1A inducers have anticarcinogenic properties [23–25]. Nevertheless, an insufficient understanding of molecular mechanisms underlying this interference limits the clinical use of such substances. Thus, identification of novel molecules suppressing the PAH-induced *CYP1A* activation and elucidation of the negative regulation of *CYP1A* may facilitate the development of novel compounds that prevent tumor formation.

Previously we have shown that menadione (vitamin K_3_, 2-methyl-1,4-naphthoquinone) is a weak inducer of CYP1A in experimental animals [26]. The aims of the present study were to evaluate the effects of menadione on BP-induced activity of CYP1A *in vitro* and *in vivo* and to identify the underlying molecular mechanisms. We found that menadione inhibits CYP1A1 activity *in vitro* by a noncompetitive mechanism. *In vivo*, menadione suppressed BP-induced CYP1A activation at the transcriptional level. This effect was accompanied by a decrease in the binding of AhR-ARNT to XRE and by an increase in DNA-binding activities of Oct-1 and C/EBP.

## Materials and Methods

### Reagents

Acrylamide, tris(hydroxymethyl)aminomethane, NADPH, ammonium persulfate, bovine serum albumin, ethyleneglycoltetraacetic acid (EGTA), poly[dI-dC], spermidine, dithiothreitol (DTT), phenylmethanesulfonylfluoride (PMSF), naphthol AS-MX phosphate, Fast Red Salt, Ponceau S, an anti-mouse biotinylated antibody, ExtrAvidin-Alkaline Phosphatase, and menadione were purchased from Sigma−Aldrich (USA); Protan Nitrocellulose Transfer Membrane from Schleicher & Schuell (Germany); spermine from ICN (USA); ammonium sulfate from Helicon (Russia); caproic acid from AppliChem (Germany); N,N′-methylenebisacrylamide, sodium dodecyl sulfate (SDS), dimethyl sulfoxide (DMSO), HEPES, and TEMED from Serva GmbH (Germany); 2-mercaptoethanol and Tween 20 from Ferak Berlin (Germany); MgCl_2_ from Janssen Chimica (Belgium); glycine and ethylenediaminetetraacetic acid (EDTA) from Merck (Germany); the TRI-Reagent and RNAsecure Reagent from Ambion, Inc. (USA); 7-ethoxyresorufin, 7-methoxyresorufin, M-MuLV reverse transcriptase, and RNasin from Promega Corporation (USA); the PCR kit qPCRmix-HS from Evrogen (Russia); glycerol from Panreac Quimica S.A.U. (Spain); α^32^P-ATP from Amersham Pharmacia Biotech (USA); Klenow enzyme and sucrose from Medigen (Russia); oligonucleotides (primers) for analysis of the rat genes *Cyp1a1*, *Cyp1a2*, *AhR*, *AhRR*, *ARNT*, and *Gapdh* as well as XRE3, NRE (negative regulatory element), C/EBP, and random hexanucleotide primers were from Syntol (Russia) or Medigen (Russia). All other chemicals were of analytical grade.

### Animals

Experiments were performed on male Wistar rats (weighing 100–200 g) from the stock maintained at the Animal Facility of the Institute of Cytology and Genetics, SB RAS, (Novosibirsk, Russia). The use of the experimental animals and experimental procedures were approved by the Animal Care Committee of the Institute of Molecular Biology and Biophysics, SB RAMS (Novosibirsk, Russia). The number of animals per group was estimated on the basis of the data from a pilot experiment to achieve statistical power of 0.8. The animals were housed in plastic cages in groups of five under standard conditions (12:12 h light/dark regimen; food and water available *ad libitum*). The compounds to be administered were dissolved in vegetable oil. The rats were injected with BP intraperitoneally (25 mg/[kg body weight]) once a day for three days, or received menadione (15 mg/kg) for four days per os, or received both BP (25 mg/kg) for three days and menadione (15 mg/kg) for four days. The control group received vegetable oil. The dose and duration of menadione administration were chosen on the basis of our previous data [27] to achieve a maximal effect on CYP1A enzymes. All rats were examined and weighed daily. No signs of toxicity or pain were observed in the treatment groups. The rats were anesthetized with ethyl ether and decapitated 24 h after the last administration of menadione.

### Preparation of rat liver microsomes

Rat liver microsomes were prepared by differential ultracentrifugation [28]. The livers were transcardially perfused with a cold buffer consisting of 1.15% KCl and 20 mM Tris-HCl (pH 7.4), dissected, and homogenized in the same buffer. Liver homogenates were centrifuged for 20 min at 10,000 × *g*, and the resulting supernatants were centrifuged for 60 min at 105,000 × *g*. The final pellets were resuspended in 0.1 M KH_2_PO_4_ buffer (pH 7.4) containing 20% of glycerol. Protein concentrations were measured by the Lowry method [29], with bovine serum albumin as a standard.

### Assays of enzymatic activity

Selective activities of the CYP1A isoforms 7-ethoxyresorufin-O-deethylase (CYP1A1) and 7-methoxyresorufin-O-demethylase (CYP1A2) were measured by the spectrofluorimetric method described by Burke and colleagues [28].

NADPH reductase activity was measured spectrophotometrically with cytochrome c as a substrate [30]. Glutathione S-transferase (GST) activity was quantified spectrophotometrically with 2,4-dinitrochlorobenzene as a substrate as described by Habig [31]. Alanine transaminase (ALT) and aspartate transaminase (AST) activities were measured on an AU480 Chemistry System (Beckman Coulter, USA) by the kinetic method according to the manufacturer’s instruction manual.

### The malondialdehyde (MDA) assay

The level of MDA was measured by the method based on the reaction with the thiobarbituric acid described by Andreeva [32]. Briefly, 200 μL of hepatic microsomes was mixed with 500 μL of 50 mM Tris-HCl (pH 7.2) and 500 μL of 15% trichloroacetic acid. The resulting mixture was centrifuged at 1500 × *g* for 10 min. The supernatant was collected and mixed with 0.75% thiobarbituric acid (1:1), and these samples were incubated in a water bath at 100°C for 15 min, then placed on ice for 5 min, and centrifuged at 1000 × *g* for 10 min. After that, absorbance at 532 nm (A_532_) of the resulting supernatant was measured, with 580 nm as the reference wavelength (used for baseline correction). MDA quantity was calculated as C_(MDA)_ = (A_532_ − A_580_) ÷ K, where K is a millimolar extinction coefficient equal to 155 mM^−1^ cm^−1^.

### RNA isolation, reverse transcription, and real-time PCR

Total RNA was isolated using the TRI-Reagent kit (Ambion). The RNA pellets were dissolved in 1 mM sodium citrate buffer (pH 6.5) containing 1× RNAsecure Reagent (Ambion). Contaminating DNA was digested with RNase-free DNase (Promega, USA) under the following conditions: 3 μg of total RNA, 3 units (U) of RQ1 RNase-free DNase, reaction buffer (40 mM Tris-HCl pH 8.0, 10 mM MgSO_4_, and 1 mM CaCl_2_), and 20 U of RNasin. Reverse transcription was performed using M-MLV Reverse Transcriptase (Promega, USA). The reaction mixture (25 μL) contained 500 ng of total RNA, reaction buffer (50 mM Tris-HCl pH 8.3, 75 mM KCl, 3 mM MgCl_2_, and 10 mM DTT), 1 mM dNTPs, 200 U of M-MuLV reverse transcriptase, 4 μg of random hexamer primers, and 25 U of RNasin.

The sequences of PCR primers are presented in Table 1. *Gapdh* was used as a reference gene. The gene expression was evaluated on an iCycler CFX96 real-time PCR detection system (Bio-Rad Laboratories, USA) according to the TaqMan principle. The reaction mixture contained the qPCRmix-HS buffer (Evrogen, Russia), 100 nM probe, 400 nM of the forward and reverse primers, and 1000 ng of cDNA. The reaction was carried out under the following conditions: initial denaturation at 95°C for 3 min, then 40 cycles of denaturation at 95°C for 15 s and annealing/extension at 60°C for 30 s. All the samples were analyzed in triplicate in two independent experiments. Relative cDNA levels of the genes under study and of the reference gene were calculated in the iCycler CFX96 real-time PCR software on the basis of standard curves built for each experiment with serial dilutions (1:3 to 1:27) of a cDNA standard (prepared by mixing cDNA from all samples) [33, 34]. For each sample, the amount of cDNA of a gene in question was normalized to the reference gene.

**Table 1:** Primer sequences for analysis of gene expression.

Gene		Sequence
***Cyp1a1***	Forward	5′-CCAAACGAGTTCCGGCCT-3′
***Cyp1a1***	Reverse	5′-TGCCCAAACCAAAGAGAATGA-3′
***Cyp1a1***	Probe	5′(FAM)-TTCTCACTCAGGTGTTTGTCCAGAGTGCC-(BHQ1)3′
***Cyp1a2***	Forward	5′-CGCCCAGAGCGGTTTCTTA-3′
***Cyp1a2***	Reverse	5′-TCCCAAGCCGAAGAGCATC-3′
***Cyp1a2***	Probe	5′(FAM)-CAATGACAACACGGCCATCGACAAG-(BHQ1)3′
***AhR***	Forward	5′-TGGACAAACTCTCCGTTCTAAGG-3′
***AhR***	Reverse	5′-GATTTTAATGCAACATCAAAGAAGCT-3′
***AhR***	Probe	5′(FAM)-CAGCGTCACGTACCTGAGGGCCA-(BHQ1)3′
***ARNT***	Forward	5′-TGGACCCTGTTTCCATGAATAG-3′
***ARNT***	Reverse	5′-TGCAGTGGACTACCACAAAG-3′
***ARNT***	Probe	5′(FAM)-FAM-ATGCAGGAATGGACTTGGCTCTGT-(BHQ1)3′
***AhRR***	Forward	5′-CAGTGCTGCCTTCTGTGACTGA-3′
***AhRR***	Reverse	5′-CTCCATTGCTCCTTCCTGCTAA-3′
***AhRR***	Probe	5′(FAM)-TTCTTGAAGGCAAAGCACACAGGGCAGAC-(BHQ1)3′
***Gapdh***	Forward	5′-CAAGGTCATCCATGACAACTTTG-3′
***Gapdh***	Reverse	5′-GGGCCATCCACAGTCTTCTG-3′
***Gapdh***	Probe	5′(FAM)-ACCACAGTCCATGCCATCACTGCCA-(BHQ1)3′

### Sodium dodecyl sulphate polyacrylamide gel electrophoresis (SDS-PAGE) and immunoblotting

SDS-PAGE was carried out according to the Laemmli method [35]. For detection of CYP1A, 0.5 μg of microsomal proteins (from rats treated with either BP or BP and menadione) or 80 μg of protein (from control rats) was loaded onto each lane of a 10% polyacrylamide gel, separated at 20 mA, and transferred to a nitrocellulose membrane by means of Fastblot B34 (Biometra, Germany). Efficiency of the transfer was confirmed by Ponceau S staining. The membrane was blocked in 5% nonfat dry milk in 1× TBS buffer (20 mM Tris-HCl pH 7.5, 150 mM NaCl) containing 0.05% Tween 20 (TBS-T), overnight at 4°C, and was then incubated for 1 h at 37°C on a shaker. The membrane was washed three times with TBS-T and was then incubated with antibodies against rat CYP1A1 and CYP1A2 [36] for 1.5 h at room temperature. After that, the membrane was washed three times with TBS-T and incubated with an alkaline phosphatase-conjugated secondary antibody for 1 h. The membranes were washed three times with TBS-T. The bound antibodies were visualized using naphthol AS-MX phosphate and Fast Red Salt.

### Preparation of nuclear protein extracts

Nuclear extracts from the liver of the experimental animals were prepared according to the method of Gorski and coauthors [37] with modifications described by Shapiro et al. [38]. Briefly, the livers were transcardially perfused with the buffer consisting of 1.15% KCl and 20 mM Tris-HCl pH 7.4, cut into fragments, and homogenized in sucrose buffer (10 mM HEPES pH 7.6, 25 mM KCl, 0.15 mM spermine, 0.5 mM spermidine, 1 mM EDTA, 2.05 M sucrose, and 10% glycerol). The homogenates were layered on top of 5 mL of sucrose buffer and centrifuged in a SW28 rotor (Optima L-90K Ultracentrifuge, Beckman Coulter, USA) at 24,000 rpm for 40 min. The pelleted nuclei were resuspended in 4 mL of lysis buffer (10 mM HEPES pH 7.6, 100 mM KCl, 3 mM MgCl_2_, 0.3 M EDTA, 1 mM DTT, 10% glycerol, and 0.1 mM PMSF) and lysed by the addition of 0.4 mL of a supersaturated (NH_4_)_2_SO_4_ solution. Chromatin was precipitated by centrifugation in the SW65 rotor at 38,600 rpm for 90 min. Nuclear proteins were precipitated by (NH_4_)_2_SO_4_ (0.252 g per milliliter of the protein solution) and collected by centrifugation in the SW65 rotor at 36,200 rpm for 20 min. The resulting pellets were dissolved in 0.2–0.5 mL of dialysis buffer (25 mM HEPES pH 7.6, 80 mM KCl, 0.1 mM EDTA, 0.2 mM EGTA, 1 mM DTT, 10% glycerol, and 0.1 mM PMSF). The nuclear extracts were dialyzed against 100 volumes of the buffer three times for 30–45 min each, split into aliquots, and stored at −70°C. Protein concentration was determined by the Bradford method [39]. Bovine serum albumin served as a standard.

### The electrophoretic mobility shift assay (EMSA)

To assess the binding of nuclear proteins to the specific DNA sequences, we used the following oligonucleotides: for XRE3 sequences, forward 5′-TGCACGGAGTTGCGTGAGAAGAGCCATGCA-3′ and reverse 3′-ACGTgcctcaacgcactcttctcggtACGT-5′ [40]; for C/EBP consensus sequences, forward 5′-TGCAGATTGCGCAATCTGCA-3′ and reverse 3′-ACGTCTAACGCGTTAGACGT-5′ [41]; for C/EBP mutant sequences, forward 5′-TGCAGAGACTAGTCTCTGCA-3′ and reverse 3′-ACGTCTCTGATCAGAGACGT-5′ [41]; for Oct-1 consensus sequences, forward 5′-TGCATGTCGAATGCAAATCACTAGAATGCA-3′ and reverse 3′-ACGTACAGCTTACGTTTAGTGATCTTACGT-5′ [42]; and for Oct-1 mutant sequences, forward 5′-TGCATGTCGAATGCAAGCCACTAGAATGCA-3′ and reverse 3′-ACGTACAGCTTACGTTCGGTGATCTTACGT-5′ [42]. The oligonucleotides were radioactively labeled using a standard protocol [43]. Briefly, 20 pmol of oligonucleotides for XRE3 sequences were incubated with 20 μCi of α^32^P-dATP and 2 U of Klenow polymerase in a buffer consisting of 50 mM Tris-HCl pH 7.6, 10 mM MgCl_2_, and 5 mM DTT. The oligonucleotides were purified from unbound α^32^P-dATP on G-50 spin columns or DEAE filters. Nucleoprotein complexes were formed under the following conditions: 1× DNA binding buffer (25 mM HEPES pH 7.6, 80 mM KCl, 0.1 mM EDTA, 1 mM DTT, 10% glycerol, 4–5 μg of protein of nuclear extracts [depending on the oligonucleotide being tested], 0.83 μg of salmon sperm DNA, and a ^32^P-labeled DNA probe [400–1000 cpm/reaction]). All components of the reaction mixture except for the labeled oligonucleotide were mixed in a total volume of 11 μL and preincubated on ice for 10 min to block nonspecific binding. After that, a ^32^P-labeled DNA probe was added, and the samples were incubated at room temperature for 10 min. To assess specificity of the DNA-protein complexes, either mutant oligonucleotides were used or a 20-, 50-, or 75-fold excess of an unlabeled oligonucleotide was added to the reaction mixture. The resulting DNA-protein complexes were analyzed by electrophoresis in a 4% nondenaturing polyacrylamide gel in 0.5× TBE buffer (44.5 mM Tris base, 44.5 mM boric acid, and 1 mM EDTA). After the electrophoresis, the gel was dried, and the DNA-protein complexes were visualized by autoradiography. Each experiment was repeated twice with samples from three rats.

### *In vitro* evaluation of menadione’s effects on CYP1A1 activity

Immediately before measurement of the CYP1A1 activity, menadione was added to the microsomes at a final concentration 0.977, 1.955, 4.614, 7.775, 9.775, 14.662, 19.549, 24.44, or 29.324 μM. Each concentration of menadione was analyzed three times. The inhibition constant (K_i_) was determined by the graphical method as modulus of the value of inhibitor concentration at the point of intersection of the X-axis and a plot of 1/(reaction rate) versus 1/(substrate concentration) in the presence of different menadione concentrations [44]. To ensure correct calculation of the reaction rate, the remaining 7-ethoxyresorufin-O-deethylase (EROD) activity in the liver microsomes was tested once every six measurements. To determine the type of inhibition, we used the graphical method in which 1/(rate of reaction) was plotted against 1/(ethoxyresorufin concentration) in the absence or presence of different concentrations of menadione [44]. The appearance of the plot indicated a noncompetitive mechanism of inhibition of CYP1A1 activity by menadione *in vitro*.

### Statistical analysis

All calculations were performed in the Statistica software package (StatSoft, Inc., USA). Differences between groups were assessed by Student’s *t* test, the Mann–Whitney rank sum test, or analysis of variance (ANOVA) with the Newman–Keuls *post hoc* test.

## Results

### The effect of menadione on BP-induced CYP1A1 activity *in vitro*

**Fig 1:**
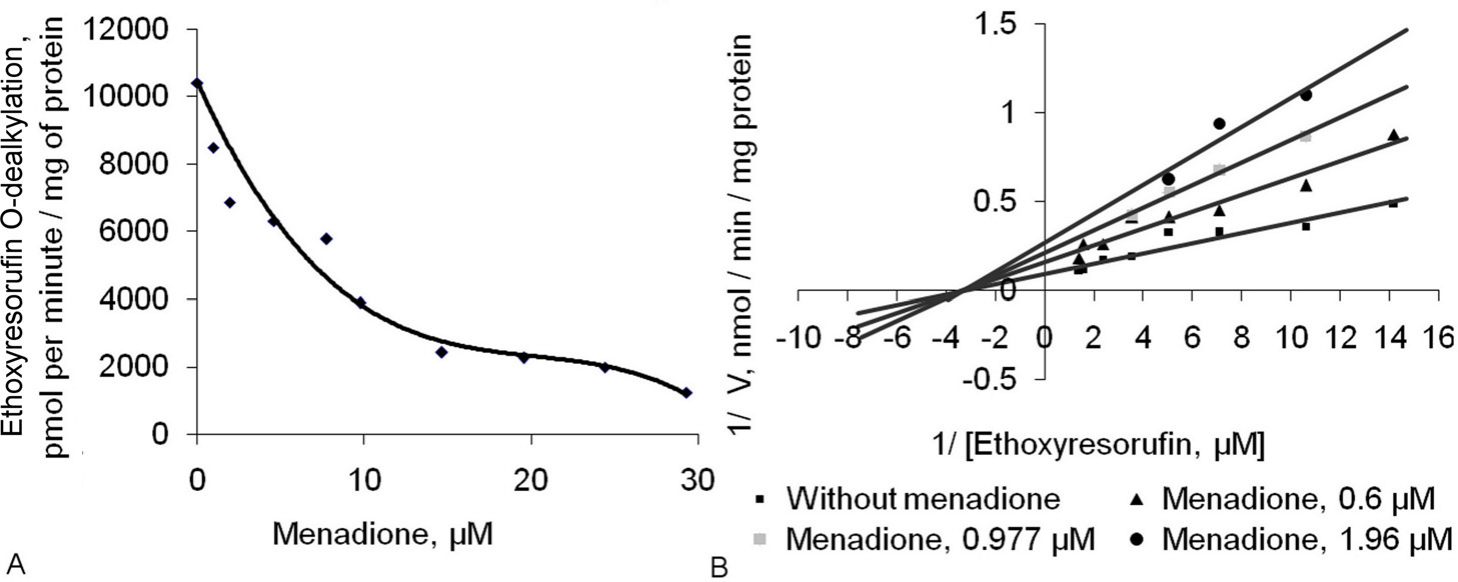
Menadione inhibits CYP1A1 activity *in vitro* via a noncompetitive mechanism. (A) Menadione inhibits CYP1A1 activity *in vitro* in the hepatic microsomes of benzo(α)pyrene-treated rats by a (B) noncompetitive mechanism with K_i_ = 3.24 μM. All measurements were performed in duplicate in three independent experiments. CYP1A1 activity was measured by means of the rate of O-dealkylation of ethoxyresorufin as described by Burke and colleagues [28].

To study the effect of menadione on BP-induced CYP1A1 activity *in vitro*, we added various concentrations of menadione to the hepatic microsomes of rats treated with BP. The results are shown in Fig 1. Addition of 1–29 μM menadione to the hepatic microsomes of BP-treated rats caused a dose-dependent decrease of CYP1A1 activity (Fig 1A). Menadione inhibited the CYP1A1 *in vitro* noncompetitively, with K_i_ = 3.24 μM (determined graphically; Fig 1B).

### Activities of hepatic enzymes, weight gain, and lipid peroxidation in rats treated with BP or BP and menadione

**Fig 2:**
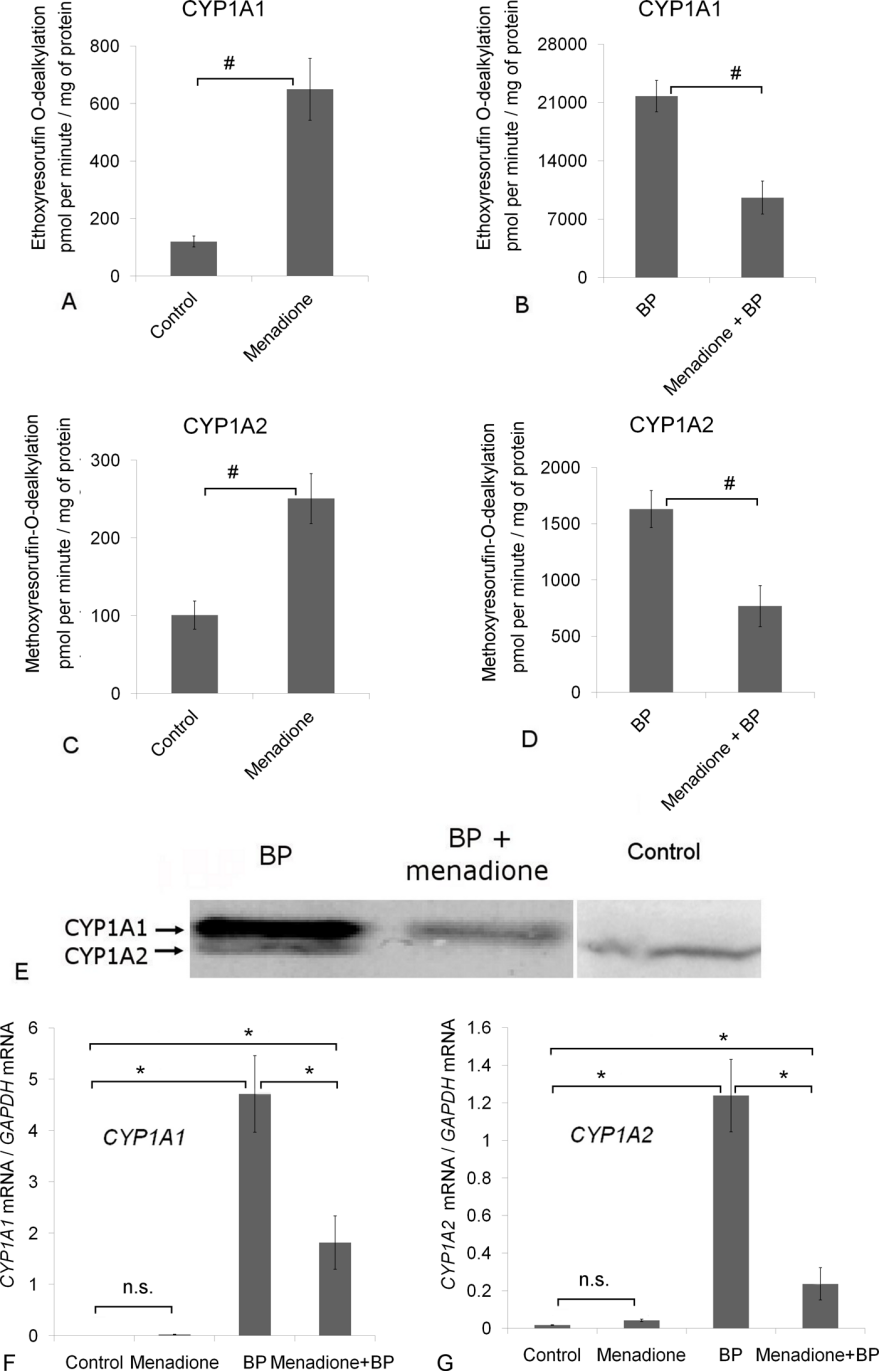
Menadione inhibits benzo(α)pyrene (BP)-induced CYP1A activity *in vivo* via a transcriptional mechanism. (A, C) Administration of menadione increases activity of CYP1A1 and CYP1A2. (B, D) Coadministration of BP and menadione suppresses activity of CYP1A1 and CYP1A2 and (E) their protein and (F, G) mRNA expression that was induced by BP. Rats received BP at 25 mg/(kg body weight) once a day for three days, menadione (15 mg/kg for four days), or both BP (25 mg/[kg body weight] for three days) and menadione (15 mg/kg for four days). The control group received vegetable oil. The data are presented as mean ± SEM (n = 4 to 8); *p < 0.05 according to ANOVA with the Newman–Keuls *post hoc* test, #p < 0.05 according to Student’s *t* test. CYP1A1 activity was measured by means of the rate of ethoxyresorufin O-dealkylation, whereas CYP1A2 activity by means of the rate of methoxyresorufin-O-dealkylation as described by Burke and colleagues [28]. Panel C: to visualize CYP1A2 on all lanes, we loaded the samples (corresponding to the treatment with BP and BP+menadione) at 0.5 μg of total protein per well and the control sample at 80 μg of total protein per well.

We previously demonstrated that menadione is a weak transcriptional inducer of CYP1A [26]. In the present study, we administered a combination of BP and menadione to rats to determine the effect of coadministration of a weak CYP1A inducer and a strong one. In line with results of other studies [4, 11–13], there was a significant increase in CYP1A1/2 activity in the liver of the rats treated with menadione or BP (Fig 2A–2D). Coadministration of BP and menadione caused an approximately twofold decrease in CYP1A1/2 activity (Fig 2B and 2D) in comparison with the rats injected with BP alone.

Activity of another enzyme that is partly controlled by AhR [45]—glutathione S-transferase (GST)—was also reduced in the liver of the rats treated with both BP and menadione, as compared to the rats treated with BP alone (S1 Fig). At the same time, menadione affected neither NADPH reductase (a key enzyme in the electron transfer chain; S2 Fig) nor lipid peroxidation, judging by the results of MDA quantification (S3 Fig).

No signs of general toxicity or acute adverse effects were detected in the treatment groups. The weight of the experimental animals remained unchanged during the study period (S4A Fig). Menadione administration did not alter liver function either: activity of the liver enzyme ALT in the serum of rats treated with menadione or both BP and menadione remained the same as that in control animals (S4B Fig); activity of AST increased in response to BP by 28%, and a similar change was seen in the group treated with both BP and menadione (S4C Fig).

Taken together, these results indicate that the observed reduction in BP-induced CYP1A activities may be caused by a decrease in the *CYP1A* mRNA level (as a result of either transcriptional downregulation or mRNA destabilization) and is not due to disruption of the electron transfer chain, oxidative damage to the enzyme, liver damage, or acute toxicity.

### Molecular mechanisms of the suppression of BP-induced CYP1A activity by menadione

To study the possible mechanism of menadione-mediated suppression of BP-induced CYP1A activity *in vivo*, we assessed the protein and mRNA levels of these enzymes in the liver of the rats by western blotting and quantitative PCR (qPCR)/RT-PCR. As expected, in the hepatic microsomes of untreated (control) rats, the CYP1A1 protein was undetectable, whereas the CYP1A2 protein was detectable. BP and menadione elevated the levels of the CYP1A1 and CYP1A2 proteins (Fig 2E and [26]). In the liver of the rats treated with both BP and menadione, the CYP1A protein levels were lower in comparison with the group receiving BP alone (Fig 2E), but still much higher than in the liver of the untreated animals.

As expected [1, 2], BP administration led to a significant increase in *CYP1A1* and *CYP1A2* mRNA levels according to both qPCR (Fig 2F and 2G) and RT-PCR (S5 Fig and S1 Text). In contrast to previous reports [26, 46], we observed only a statistically insignificant trend for an increase in the level of mRNA of these genes in response to menadione, according to qPCR (Fig 2F and 2G). In agreement with the data on enzymatic activities (Fig 2B and 2E) and western blotting results (Fig 2E), there was a reduction in mRNA levels of *CYP1A1* and *CYP1A2* in the liver of the rats receiving both BP and menadione in comparison with the BP-treated animals. Combined administration of menadione and BP caused a 2.6-fold decrease in the *CYP1A1* mRNA level (Fig 2F) and a 5.3-fold decrease in the *CYP1A2* mRNA level (Fig 2G) in comparison with BP treatment. These data indicate that menadione-mediated inhibition of BP-induced CYP1A *in vivo* takes place at the transcriptional level. It appears that the inhibitory properties of menadione towards CYP1A1 enzymatic activity (Fig 1) may contribute to the overall inhibitory effect but do not represent the primary mechanism of menadione-driven downregulation of the BP-induced CYP1A1 activation *in vivo*.

**Fig 3:**
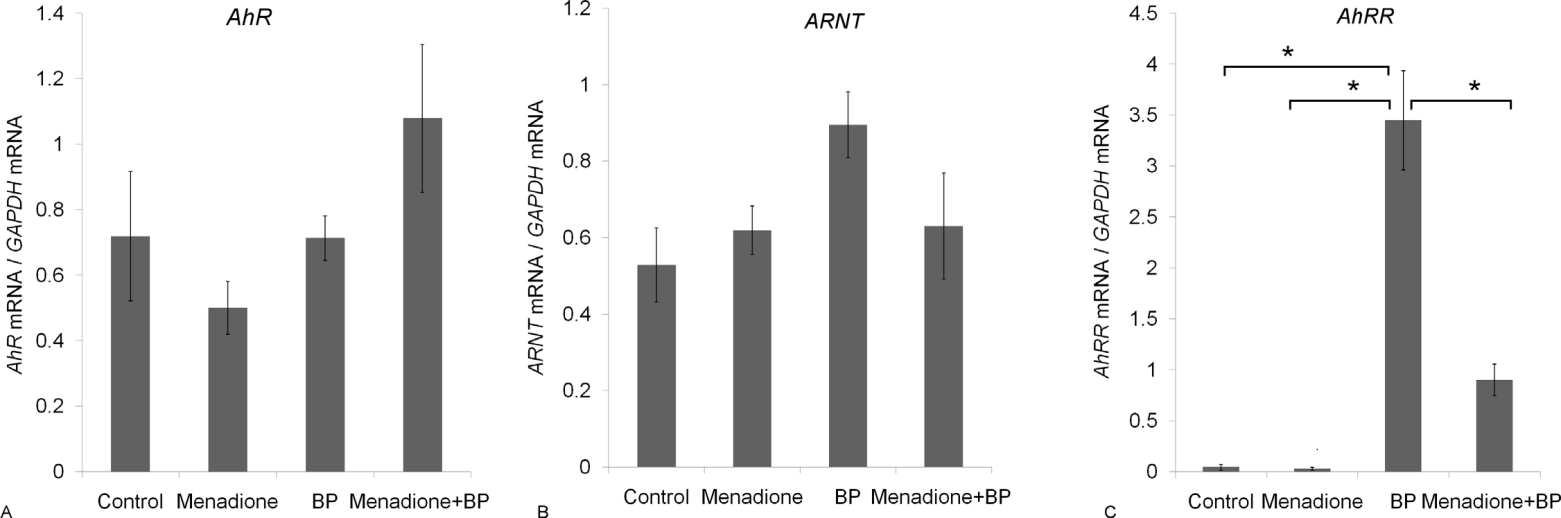
Menadione does not change mRNA expression of *AhR* and *ARNT* but reduces benzo(α)pyrene (BP)-induced *AhRR* expression. Menadione does not influence the expression of genes *AhR* (panel A) and *ARNT* (panel B), but reduces the mRNA level of AhR repressor (*AhRR*) (panel C) that is increased by BP. Rats received BP at 25 mg/(kg body weight) once a day for three days, menadione (15 mg/kg for four days), or both BP (25 mg/[kg body weight] for three days) and menadione (15 mg/kg for four days). The control group received vegetable oil. The data are presented as mean ± SEM (n = 4 to 5); *p < 0.05 according to ANOVA with the Newman–Keuls *post hoc* test.

Next, we analyzed mRNA levels of *AhR*, *ARNT*, and AhR repressor (*AhRR*) because the quantities of these proteins are known to influence *CYP1A* expression [2, 47–49]. As shown in Fig 3A and 3B, neither *AhR* nor ARNT were affected significantly by any of the treatments tested in the present study. The mRNA level of *AhRR* (Fig 3C) was below the detection limit in the liver of the untreated animals. BP administration robustly increased *AhRR* expression in comparison with the untreated and menadione-treated animals. On the other hand, coadministration of menadione and BP was accompanied by a reduction in the *AhRR* mRNA level in comparison with the group receiving BP alone. The above results suggest that the menadione-driven inhibition of BP-induced CYP1A activation cannot be explained either by downregulation of AhR/ARNT or by upregulation of AhRR expression.

**Fig 4:**
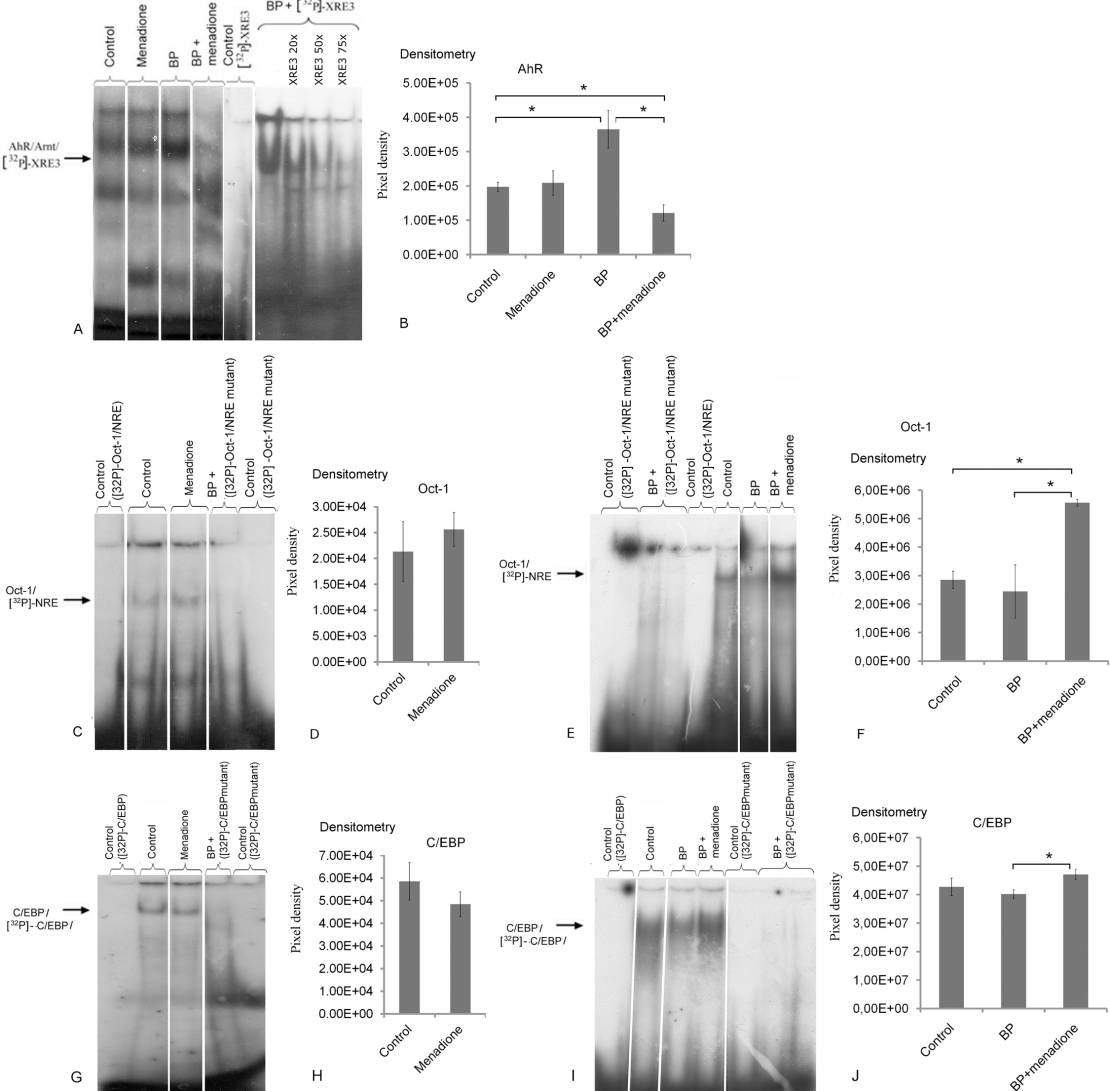
Menadione changes DNA-binding activity of some nuclear proteins from the rat liver (AhR/ARNT, Oct-1, and C/EBP). (A, B) EMSA analysis of binding of AhR/ARNT to XRE3, (C–F) Oct-1 to NRE, and (G–J) C/EBP to the C/EBP response element in the nuclear extracts from the liver of untreated rats (control) and the rats treated with menadione (15 mg/kg for four days), benzo(α)pyrene (BP; 25 mg/kg), or both BP (25 mg/[kg body weight] for three days) and menadione (15 mg/kg for four days). The specificity of the bands was confirmed by means of mutated oligonucleotides or an excess of an unlabeled oligonucleotide. The density of the bands was measured in pixels using Totallab software. The data are presented as mean ± SEM (n = 3); *p < 0.05 according to ANOVA with the Newman–Keuls *post hoc* test.

We then tested functional activity of the AhR–ARNT complex by an EMSA. Treatment with BP, but not menadione, increased DNA-binding activity of AhR in the nuclear extracts from the rat liver (Fig 4A and 4B). Coadministration of menadione and BP caused a pronounced decrease in the intensity of the bands corresponding to the ^32^P-labeled XRE3–AhR–ARNT complex in comparison with the treatment with BP or menadione only. The dose-dependent decrease of the intensity of the respective band in the presence of the 20-, 50-, or 75-fold excess of an unlabeled oligonucleotide is indicative of the specificity of the binding (Fig 4A).

Because transcription factors Oct-1 and C/EBP are involved in negative regulation of CYP1A [3, 4], we attempted to determine whether these transcription factors are involved in the menadione-driven downregulation of BP-induced CYP1A activity. As shown in Fig 4C–4J, no changes in DNA-binding activity of these proteins were observed in the liver of the experimental animals in response to either menadione or BP (Fig 4C–4J). Coadministration of menadione and BP increased the DNA-binding activity of Oct-1 (Fig 4E and 4F) and C/EBP (Fig 4I and 4J) in comparison with the control group or group receiving BP treatment alone. Specificity of the binding was confirmed by means of mutant oligonucleotides. Thus, it is likely that menadione promotes binding of the proteins Oct-1 and C/EBP to the CYP1A promoter and thereby downregulates the BP-induced CYP1A expression.

**Fig 5:**
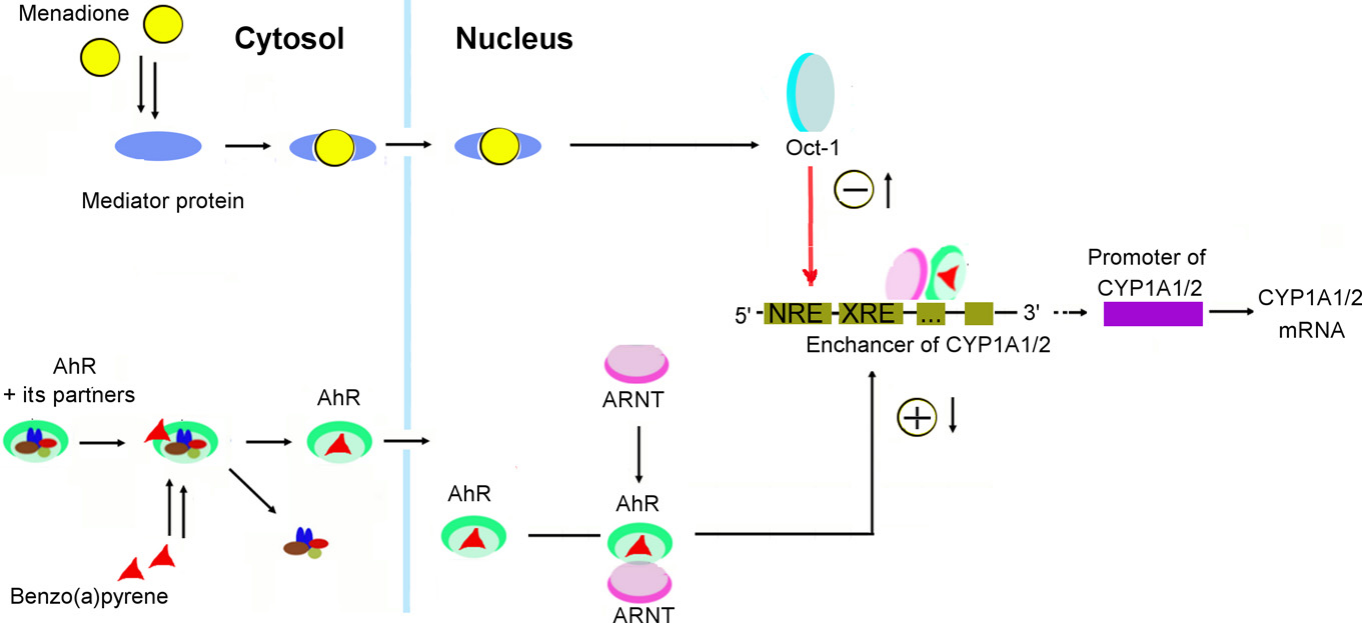
Possible mechanisms of the menadione-driven inhibition of the benzo(α)pyrene (BP)-induced AhR-dependent signal transduction pathway. Menadione decreases DNA-binding activity of the AhR–ARNT complex thus reducing positive input, and at the same time, activates Oct-1, which inhibits expression of AhR-dependent genes. red triangle—Benzo(α)pyrene, green oval with brown, blue, yellow and red ovals inside—inactive AhR in complex with its protein partners, green oval with a triangle inside—AhR activated by benzo(α)pyrene, pink oval—ARNT, yellow circle—menadione, blue oval—mediator protein, turquoise–light gray oval—Oct-1, NRE: negative regulatory element, XRE: xenobiotic-responsive element, AhR: aryl hydrocarbon receptor, ARNT: aryl hydrocarbon receptor nuclear translocator.

Our results indicate that menadione inhibits CYP1A1 activity *in vitro* by a noncompetitive mechanism (Fig 1) and *in vivo* via suppression of *CYP1A1* gene transcription (Fig 2F). The downregulation of BP-induced *CYP1A1* mRNA is not caused by either upregulation of AhRR or reduced expression of AhR and ARNT. Instead, it is mediated by a decrease in positive input (binding of AhR to XRE) and an increase in negative input (binding of Oct-1 and C/EBP to the CYP1A1 promoter region; Fig 5).

## Discussion

Induction of CYP1A enzymes that occurs via the AhR signal transduction pathway has been well characterized previously, but negative regulation of these enzymes is poorly understood. Several studies showed that weak inducers of CYP1A can downregulate the activation of these enzymes caused by a procarcinogenic PAH via different mechanisms [11–15, 26]. In our earlier work [26], we demonstrated that menadione (a synthetic water-soluble analog of vitamin K_3_ commonly used as a therapeutic agent) is a “weak” CYP1A inducer.

In the present study, we showed that menadione can downregulate BP-induced expression of AhR-dependent genes and addressed possible mechanisms of this effect. Because menadione is known to inhibit activity of some CYPs *in vitro* [50], we first tested whether it can do the same to CYP1A1. Menadione indeed inhibited CYP1A1 activity in rat hepatic microsomes via a noncompetitive mechanism (Fig 1). In the present study, inhibition of CYP1A1 by menadione *in vitro* was found to be weaker than inhibition by the other naturally occurring CYP1A1 inhibitors such as quercetin [51], piceatannol [52], *trans*-resveratrol [52], and indolo(3,2)carbazole [53]. Nevertheless, according to our previous research on tolerable doses of this substance [27], it seemed theoretically possible to attain a sufficient concentration of menadione to achieve inhibition of BP-induced CYP1A1 activation in the rat liver.

We then proceeded to animal experiments and demonstrated that menadione indeed reduces the BP-induced activity of CYP1A in the rat liver (Fig 2B and 2D). It should be noted that *in vivo*, this effect is mediated neither by direct inhibition of enzymatic activities nor by oxidative damage (S3 Fig); this finding is in agreement with our present results (Fig 1) and previously published data [54, 55]. In addition, this interference by menadione with the action of BP is not caused by toxicity or liver damage because the liver function indicators ALT and AST remained unchanged (S4B and S4C Fig). Our results indicate that *in vivo*, menadione downregulates *CYP1A* genes via a reduction in their mRNA levels (Fig 2F and 2G). In contrast to previously published data [26], in the present study, we did not observe a statistically significant increase in the *CYP1A1* mRNA level in response to menadione. This discrepancy can be explained by the methodological differences between these studies. Previously, we collected samples 20 h after the last administration of menadione, whereas in this study, 24 h after. Upregulation of *CYP1A1* mRNA in response to an inducer may be transient, as shown for the aromatic hydrocarbon ethylbenzene [56]. In addition, menadione is rapidly excreted by kidneys [57]. Thus, the combination of these two factors can result in bell-shaped upregulation of *CYP1A1* mRNA in response to menadione; in 24 h, we might miss the peak of this upregulation.

We hypothesized that downregulation of BP-induced CYP1A1 induction by menadione can be caused by menadione’s influence on upstream regulatory proteins, because in addition to CYP1A, this compound downregulates BP-induced activity of another xenobiotic-metabolizing enzyme (GST), which is at least partially regulated by AhR (S1 Fig).

Therefore, we first measured the expression levels of *AhR*, *ARNT*, and *AhRR* because they were previously shown to modulate expression of AhR-dependent genes [58, 59]. None of the treatments influenced expression of AhR and ARNT, but menadione suppressed the BP-induced expression of AhRR. Downregulation of AhRR is not surprising because its expression (same as expression of CYP1A) is controlled by an AhR-dependent signaling pathway [60, 61]. Although some weak inducers downregulate AhR signaling via AhRR [59], our data indicate that menadione-driven suppression of the BP-induced CYP1A1 activation is not mediated by AhRR.

Next, we tested functional activity of AhR using EMSA. As expected [62], BP increased DNA-binding activity of AhR (Fig 4A and 4B). Menadione alone did not affect this activity, but when menadione was coadministered with BP, it downregulated the BP-induced binding of the AhR/ARNT heterodimer to the synthetic XRE. According to the literature, some weak AhR ligands may compete with PAH for binding to the receptor and thus form AhR–ligand complexes with a reduced ability to recruit basic transcription factors to the promoters of target genes, thereby counteracting the effects of PAH on CYP1A [63, 64]. Nonetheless, this is an unlikely mechanism behind the menadione-driven suppression of BP-induced activation of AhR-dependent genes because according to the EMSA results (Fig 4A and 4B), menadione itself fails to increase the functional activity of AhR. It should be noted that no other signal transduction pathways except the one mediated by AhR have been previously shown to upregulate CYP1A1. Our results (Figs 2 and 4) suggest that menadione increases CYP1A1 activity independently of AhR, but additional studies are necessary to clarify this question.

Because the expression of CYP1A may be inhibited by transcription factors Oct-1 and C/EBP [3, 4], we assessed the influence of menadione on functional activity of these proteins. We demonstrated that in nuclear extracts from the liver of the rats treated with both BP and menadione, DNA-binding activity of both Oct-1 and C/EBP was higher than that in the liver of the rats receiving BP alone.

Our working model regarding the suppression of the BP-induced expression of AhR-dependent genes by menadione is presented in Fig 5. Both reduction in positive regulation and enhancement of negative regulation contribute to this effect. Menadione decreases the binding of AhR/ARNT to XRE (as we saw in the nuclear extract from the liver of rats treated with BP and menadione; Fig 4A and 4B). At the same time, menadione activates the Oct-1 protein that binds to NRE in the promoter of AhR-responsive genes and downregulates their expression.

The menadione-driven inhibition of the BP-induced activities of AhR-responsive genes was only partial (~40–60% reduction). Nevertheless, comparable or even weaker suppression of the cell response to TCDD (measured using activity of CYP1A1 as a marker) by vitamin A was sufficient to decrease the liver damage in the mice treated with TCDD, according to histological analysis [15]. Taken together, these observations suggest that the menadione-driven suppression of BP-induced AhR activation and the subsequent decrease in CYP1A1 activity may have physiological and clinical significance. Many people are chronically exposed to PAHs in their lifetime because these chemicals are present in exhaust fumes of internal-combustion engines, in emissions from thermal power generators, and in tobacco smoke. Long-term exposure to PAH can lead to the development of lymphohemopoietic and gastrointestinal neoplasms and breast and lung cancers [65, 66]. Indeed, workers in the industries utilizing PAHs in technological processes—such as aluminum and coke production, iron and steel foundries, tar distillation, roofing, and road paving—are at an increased risk of lung, skin, and bladder cancers [19]. It is generally accepted that the toxicity of these chemicals is mediated by AhR [19–22]. Therefore, nutritional substances such as menadione that interfere with the action of PAHs by preventing/suppressing activation of AhR-responsive genes (such as CYP1A) *in vivo* may prevent tumor formation and development.

## Conclusions

Our results indicate that menadione suppresses BP-induced activity of AhR and transcriptionally downregulates target genes of AhR *in vivo*. This effect is accompanied by a reduction in the DNA-binding activity of the AhR/ARNT heterodimer and by enhancement of the binding of Oct-1 and CEBP to the target promotor. Because of the important role of AhR and its target genes in tumor formation, our results provide the basis for further studies of menadione in animal models of BP-induced cancer.

## Supporting Information

S1 Fig Glutathione S-transferase activity. Administration of menadione or BP increases glutathione S-transferase activity. Coadministration of BP and menadione attenuates the enhancement of glutathione S-transferase activity by BP. The data are presented as mean ± SEM (n = 4 to 9); *p < 0.05 according to ANOVA with the Newman–Keuls *post hoc* test.
(TIFF)

S2 Fig NADPH cytochrome P450 reductase activity. Menadione, BP, or coadministration of menadione and BP do not influence the NADPH cytochrome P450 reductase activity. The data are presented as mean ± SEM (n = 4 to 16).
(TIFF)

S3 Fig Malondialdehyde level. Menadione, BP, or coadministration of menadione and BP do not influence the malondialdehyde level in the liver of the experimental animals. The data are presented as mean ± SEM (n = 4 to 6).
(TIFF)

S4 Fig Body weight of the rats and transaminase activities. Menadione (15 mg/kg for 4 days), BP (25 mg/kg for 3 days), or menadione+BP do not change body weight of the rats. Menadione changes the ALT (A) and AST (B) activities in the serum of rats. In rats treated with BP, the ALT levels were lower in comparison with the group receiving menadione or BP+menadione. In the liver of the rats treated with either BP or both BP and menadione, the AST levels were higher than in the liver of untreated animals. The data are presented as mean ± SEM (n = 4 to 5); *p < 0.05 according to ANOVA with the Newman–Keuls *post hoc* test.
(TIFF)

S5 Fig ***CYP1A1*, *CYP1A1*, *AhR*, *ARNT* and *AhRR* mRNA levels measured by RT-PCR** (A, B) Coadministration of BP and menadione suppresses mRNA expression that was induced by BP. (C) Menadione does not influence the expression of the genes *AhR* and (D) *ARNT* but (E) reduces the mRNA level of AhR repressor (*AhRR*) that is increased by BP. Rats received BP at 25 mg/(kg body weight) once a day for three days, or both BP (25 mg/[kg body weight] for three days) and menadione (15 mg/kg for four days). The data are presented as mean ± SEM (n = 4 to 5); *p < 0.05 according to ANOVA with the Newman–Keuls *post hoc* test, #p < 0.05 according to Student’s *t* test.
(TIFF)

S1 Text The RT-PCR protocol. (DOCX)

## Abbreviations

AhR: aryl hydrocarbon receptor
AhRR: AhR repressor
ALT: alanine transaminase
AST: aspartate transaminase
ARNT: aryl hydrocarbon receptor nuclear translocator
BP: benzo(α)pyrene
C/EBPβ: CCAAT/enhancer-binding protein β
CYP: cytochrome P450
CYP1A1: cytochrome P450 1A1
CYP1A2: cytochrome P450 1A2
GST: glutathione S-transferase
MDA: malondialdehyde
NRE: negative regulatory element
PAH: polycyclic aromatic hydrocarbon
TCDD: 2,3,7,8-tetrachlorodibenzodioxin
XRE: xenobiotic responsive element
