# Supplementary figures and images for: Menadione Suppresses Benzo(α)pyrene-Induced Activation of Cytochromes P450 1A: Insights into a Possible Molecular Mechanism
Source: PLoS One. 2016 May 11;11(5):e0155135. doi: 10.1371/journal.pone.0155135 (PMC4864395; doi:10.1371/journal.pone.0155135)

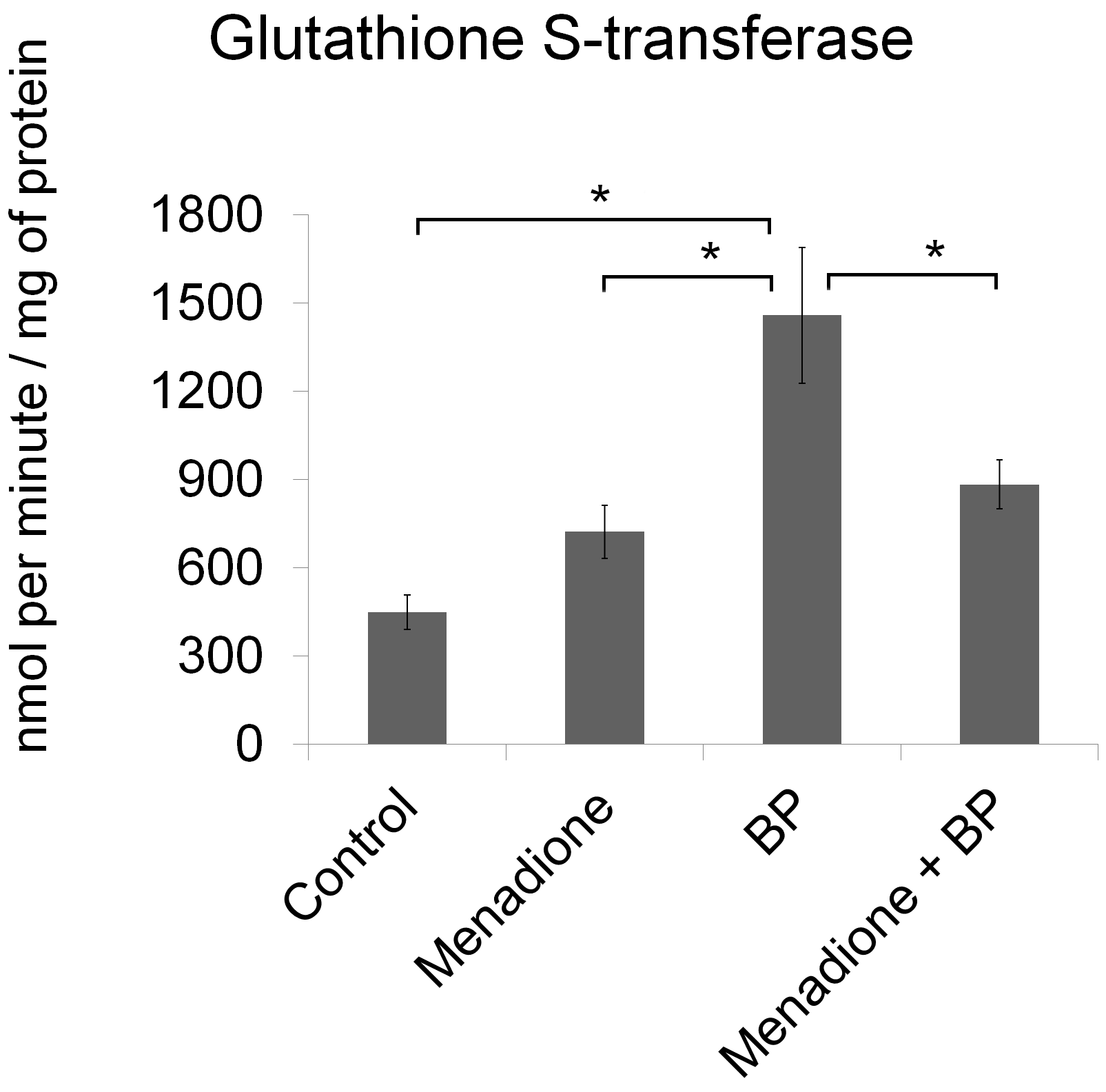

Supplement: S1 Fig — Administration of menadione or BP increases glutathione S-transferase activity. Coadministration of BP and menadione attenuates the enhancement of glutathione S-transferase activity by BP. The data are presented as mean ± SEM (n = 4 to 9); *p < 0.05 according to ANOVA with the Newman–Keuls post hoc test. (TIFF) [file pone.0155135.s001.tiff]

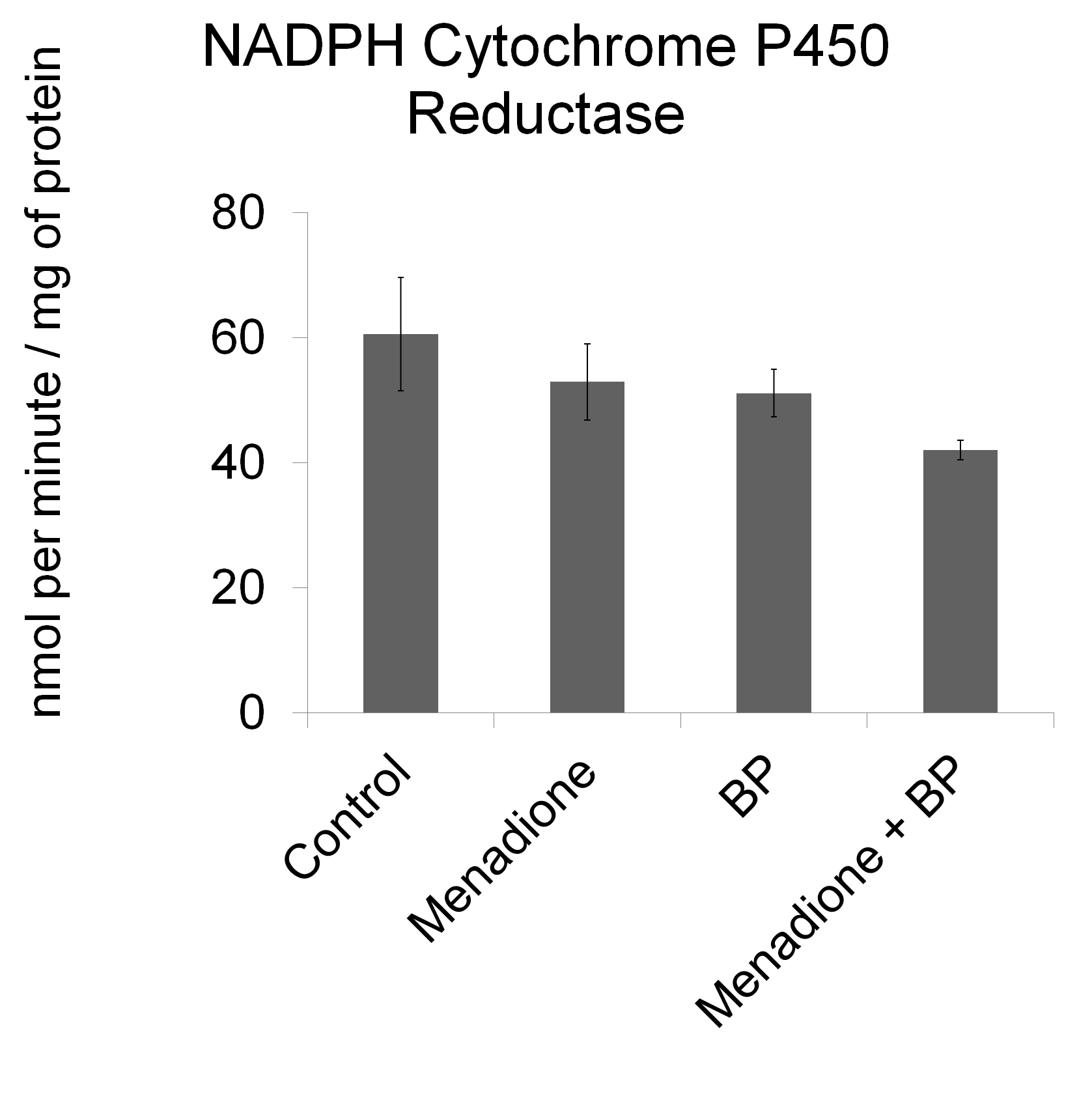

Supplement: S2 Fig — Menadione, BP, or coadministration of menadione and BP do not influence the NADPH cytochrome P450 reductase activity. The data are presented as mean ± SEM (n = 4 to 16). (TIFF) [file pone.0155135.s002.tiff]

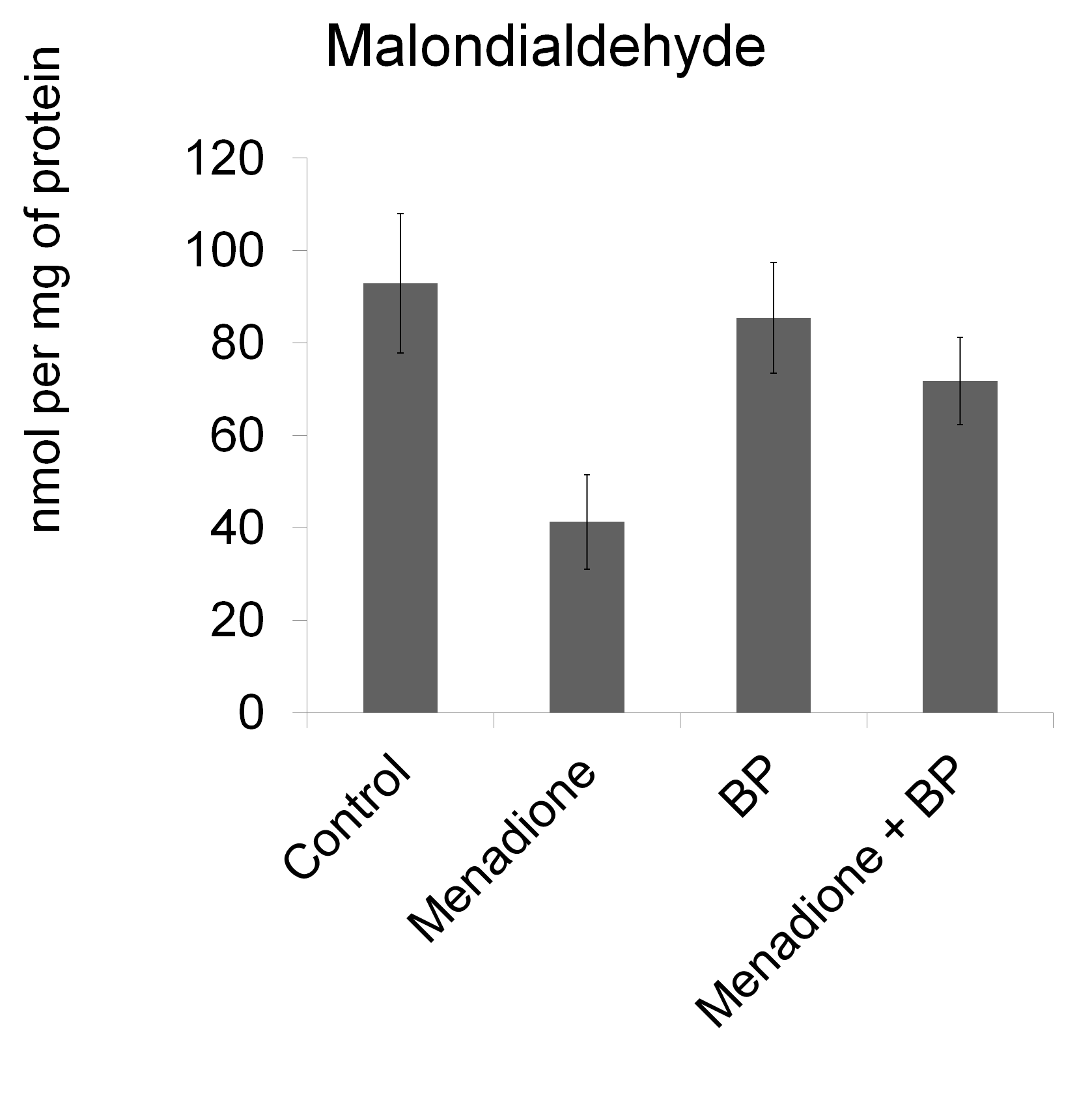

Supplement: S3 Fig — Menadione, BP, or coadministration of menadione and BP do not influence the malondialdehyde level in the liver of the experimental animals. The data are presented as mean ± SEM (n = 4 to 6). (TIFF) [file pone.0155135.s003.tiff]

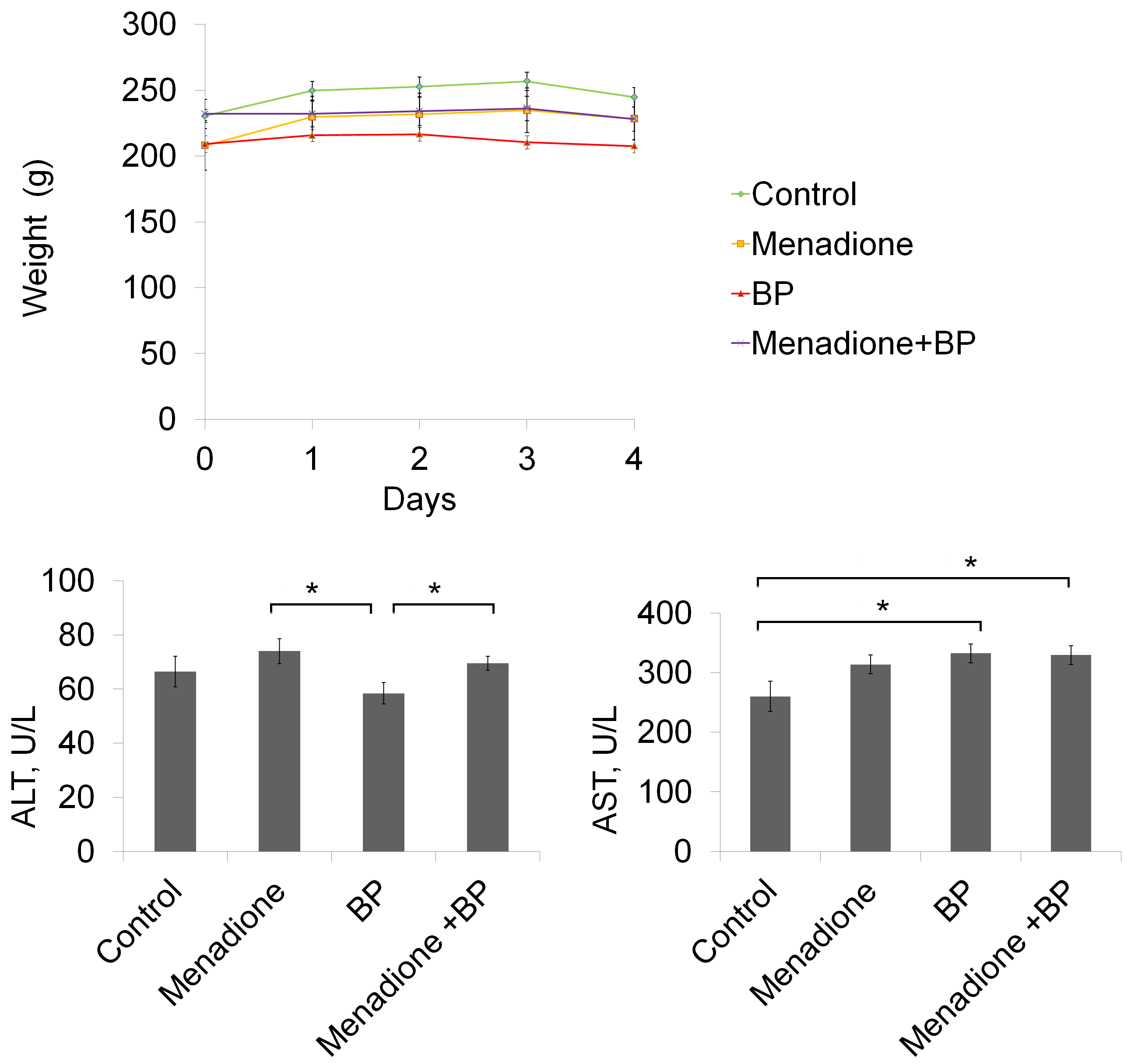

Supplement: S4 Fig — Menadione (15 mg/kg for 4 days), BP (25 mg/kg for 3 days), or menadione+BP do not change body weight of the rats. Menadione changes the ALT (A) and AST (B) activities in the serum of rats. In rats treated with BP, the ALT levels were lower in comparison with the group receiving menadione or BP+menadione. In the liver of the rats treated with either BP or both BP and menadione, the AST levels were higher than in the liver of untreated animals. The data are presented as mean ± SEM (n = 4 to 5); *p < 0.05 according to ANOVA with the Newman–Keuls post hoc test. (TIFF) [file pone.0155135.s004.tiff]

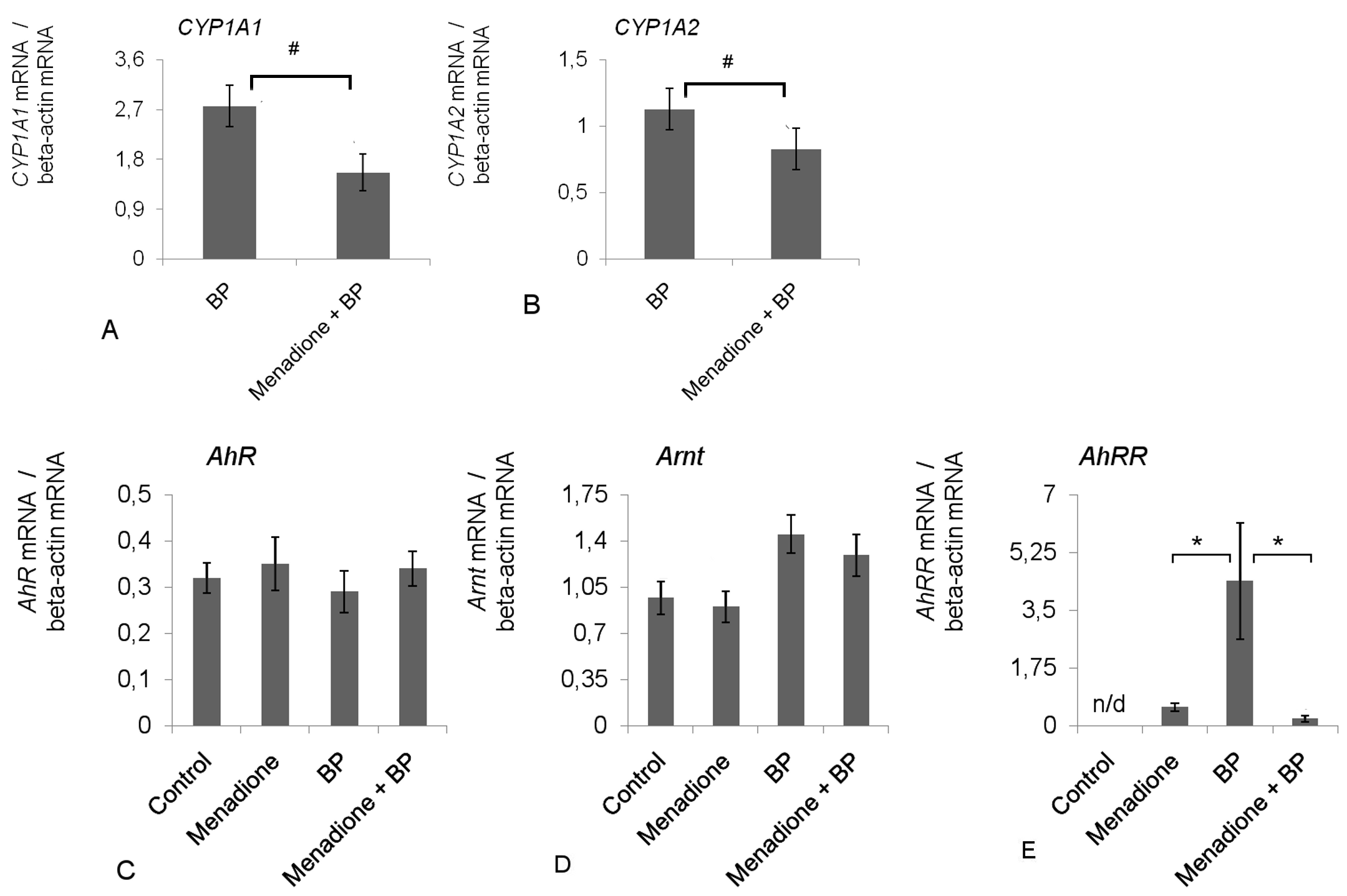

Supplement: S5 Fig — CYP1A1, CYP1A1, AhR, ARNT and AhRR mRNA levels measured by RT-PCR (A, B) Coadministration of BP and menadione suppresses mRNA expression that was induced by BP. (C) Menadione does not influence the expression of the genes AhR and (D) ARNT but (E) reduces the mRNA level of AhR repressor (AhRR) that is increased by BP. Rats received BP at 25 mg/(kg body weight) once a day for three days, or both BP (25 mg/[kg body weight] for three days) and menadione (15 mg/kg for four days). The data are presented as mean ± SEM (n = 4 to 5); *p < 0.05 according to ANOVA with the Newman–Keuls post hoc test, #p < 0.05 according to Student’s t test. (TIFF) [file pone.0155135.s005.tiff]
