# Supplementary material for: Menadione Suppresses Benzo(α)pyrene-Induced Activation of Cytochromes P450 1A: Insights into a Possible Molecular Mechanism
Source: PLoS One. 2016 May 11;11(5):e0155135. doi: 10.1371/journal.pone.0155135 (PMC4864395; doi:10.1371/journal.pone.0155135)
Supplement: S1 Text — (DOCX) [file pone.0155135.s006.docx]

**The RT-PCR protocol**

**RNA isolation and reverse transcription**

Total RNA was isolated using the VektoRNK-ekstraktsiya RNA isolation kit (ZAO Vector-Best, Russia) based on the phenol–chloroform extraction method as per the manufacturer’s protocol. The resulting RNA pellets were dissolved in 1 mМ sodium citrate buffer, pH 6.5, containing 1× RNA Secure Reagent. The RNA concentration was determined by UV spectrophotometry, and RNA integrity was verified by agarose gel electrophoresis with ethidium bromide staining. The RT reaction mixture contained 400 ng of total RNA, reaction buffer (50 mM Tris-HCl pH 8.3, 75 mM KCl, 3 mM MgCl_2_, and 10 mM DTT), 1 mM dNTPs, 200 U of M-MuLV reverse transcriptase, 4 µg of random hexamer primers, and 25 U of RNasin® in a 25-µL reaction volume. The cDNA synthesis was carried out at 37°C for 120 min.

**PCR**

We used the following PCR primers: for CYP1A1, forward 5′-CTGGTTCTGGATACCCAGCTG-3′ and reverse 5′-CCTAGGGTTGGTTACCAGG-3′, amplicon size 331 bp ([1](#_ENREF_1)); for CYP1A2, forward 5′-GCAGGTCAACCATGATGAGAA-3′ and reverse 5′-CGGCCGATGTCTCGGCCATCT-3′, amplicon size 334 bp ([2](#_ENREF_2)); for AhR, forward 5′-TCCATGTACCAGTGCCAGG-3′ and reverse 5′-ATATCAGGAAGAGGCTGGGC-3′, amplicon size 212 bp ([3](#_ENREF_3)); for ARNT, forward 5′-GTCTCCCTCCCAGATGATGA-3′ and reverse 5′-AAGAGCTCCTGTGGCTGGTA-3′, amplicon size 218 bp ([3](#_ENREF_3)); for AhRR, forward 5′-AAAGTCAGCATCCCTCCTTG-3′ and reverse 5′-CCCATCAGATCCTTTGGATG-3′, amplicon size 161 bp ([4](#_ENREF_4)); and for β-actin, forward 5′-CGTTGACATCCGTAAAGACCTCTA-3′ and reverse 5′-TAAAACGCAGCTCAGTAACAGTCCG-3′, amplicon size 290 bp ([5](#_ENREF_5)). β-Actin (housekeeping gene) was used as an internal control to confirm the proper loading and quality of cDNA in each sample. Multiplex PCR was carried out in a total volume of 20 μL in 1× PCR buffer (150 mM Tris-HCl pH 8.3, 50 mM KCl), 0.25 mM dNTPs, 0.25 μM target gene primers, 0.25 μM β-actin primers, 2 U of Taq polymerase, 2 μL of cDNA, 3.5 mM MgCl_2_ for all primers except for the AhR primers, for which 2.5 mM MgCl_2_ was used.

The PCR program started with initial denaturation at 95°C for 3 min followed by cycles of amplification under the following conditions: 94°C for 30 s; 56°C (for CYP1A1), 60°C (for CYP1A2, AhRR), 57°C (for AhR, ARNT), or 65°C (for β-actin) for 60 s; and 72°C for 60 s; and final extension at 72°C for 4 min. The number of cycles was equal to 34 (samples from control and menadione-treated rats) or 25 (samples from BP-treated rats) for CYP1A1; 28 (samples from control and menadione-treated rats) or 25 (samples from BP-treated rats) for CYP1A2; 34 for AhR, ARNT, and AhRR; 27 for β-actin. Each sample was amplified in duplicate. The PCR products were separated by electrophoresis in a 2% agarose gel in 1× TBE buffer and stained with ethidium bromide. The PCR bands were visualized using UV light, photographed by means of the DNA Analyzer Video System (Lytech, Russia), and analyzed in the TotalLab software. The level of target gene expression was expressed as a ratio of optical density corresponding to the target gene and optical density corresponding to β-actin.

**References**

1. Morris DL, Davila JC. Analysis of rat cytochrome P450 isoenzyme expression using semi-quantitative reverse transcriptase-polymerase chain reaction (RT-PCR). Biochem Pharmacol. 1996;52(5):781-92.

2. Walker NJ, Portier CJ, Lax SF, Crofts FG, Li Y, Lucier GW, et al. Characterization of the dose-response of CYP1B1, CYP1A1, and CYP1A2 in the liver of female Sprague-Dawley rats following chronic exposure to 2,3,7,8-tetrachlorodibenzo-p-dioxin. Toxicol Appl Pharmacol. 1999;154(3):279-86.

3. Lindros KO, Oinonen T, Johansson I, Ingelman-Sundberg M. Selective centrilobular expression of the aryl hydrocarbon receptor in rat liver. J Pharmacol Exp Ther. 1997;280(1):506-11.

4. Korkalainen M, Tuomisto J, Pohjanvirta R. Primary structure and inducibility by 2,3,7,8-tetrachlorodibenzo-p-dioxin (TCDD) of aryl hydrocarbon receptor repressor in a TCDD-sensitive and a TCDD-resistant rat strain. Biochem Biophys Res Commun. 2004;315(1):123-31.

5. Adams NH, Levi PE, Hodgson E. Regulation of cytochrome P-450 isozymes by methylenedioxyphenyl compounds. Chem Biol Interact. 1993;86(3):255-74.
